# Evolution of oral neoplasm in an andalusian population (Spain)

**DOI:** 10.4317/medoral.21839

**Published:** 2017-12-24

**Authors:** Rafael Flores-Ruiz, Lizett Castellanos-Cosano, Maria-Angeles Serrera-Figallo, Aida Gutierrez-Corrales, Raquel Castillo-Oyague, Jose-Luis Gutiérrez-Pere, Daniel Torres-Lagares

**Affiliations:** 1 Department of Stomatology, School of Dentistry, University of Seville; 2 Dental School, University Complutense of Madrid

## Abstract

**Background:**

Head and neck cancer are one of the most common neoplasm pathologies in humans. The aim of this study was to analyze the type, characteristics, treatment and evolution of oral neoplasm or precancerous lesion in a sample of Andalusian population (Spain) derived from the Oncology Rehabilitation Hospital Unit during a period of 20 years.

**Material and Methods:**

A retrospective descriptive study was carried out during the years 1991 and 2011 analyzing the type, characteristics, treatment and follow-up of oral neoplasm in the Oral and Maxillofacial Surgery Unit of the Universitary Hospital “Virgen del Rocío”. The inclusion criteria were patients whose underlying pathology was any type of benign or malignant neoplasm or presence of precancerous lesion that, after treatment, had been referred to the Prosthetic Rehabilitation Unit.

**Results:**

Of the initial analyzed sample of 60 patients, only 45 patients met the inclusion criteria. Of the final sample analyzed, 31 subjects were men (68.9%) and 14 women (31.1%) (*p* = 0.0169). The mean age of the sample was 57 years ± 13.83, been more frequently in older people with more than 50 years (73.3%) (*p* = 0.0169). The most common type of neoplasm was epidermoid carcinoma (64.4%). The site most frequently found in squamous cell carcinoma was the floor of the mouth (31%). The most frequent treatment modality was surgery with postoperative radiotherapy (42.2%). All patients had a minimum follow-up of 5 years, and a recurrence in this period was identified in 11.1% of the sample. Multivariate logistic regression showed a statistically significant association for the variables age (*p* = 0.0063) and smoking (*p* = 0.0434).

**Conclusions:**

Epidermoid carcinoma is the most frequent tumor in the oral cavity, where increase age and smoking are confirmed as associated risk factors.

** Key words:**Head and neck neoplasm, Squamous cell carcinoma, epidermoide carcinoma, epidemiology, risk factors.

## Introduction

Head and neck cancer are one of the most common neoplasm pathologies in humans, accounting for 3% of all types of cancers. Approximately 48% of the cases are located in the oral cavity, of which 90% correspond to squamous cell carcinoma (1,2).

This pathology affects largely of the population of middle and advanced age, where 90% occurs in people over 40 years of age, with predominance between 55 and 70 years (3). The etiology is of unknown origin although it has been shown that squamous cell carcinoma from the oropharyngeal cavity is associated with high frequency with tobacco consumption, alcohol abuse and poor nutritional status. Smoking increases by approximately six-fold the relative risk of oral cancer versus non-smokers (4).

The location of the primary tumour determines the behavior and prognosis which leads to the execution of a particular type of treatment. The treatment plan is evaluated individually in a committee specialized in head and neck tumors. The TNM classification is the most used to study and homogenize the diagnosis and treatment of tumours (5).

The main objective of cancer treatment is patient survival, although restoration of altered functions and aesthetics through reconstructive surgery and / or subsequent rehabilitation with removable prosthesis or osseointegrated implants must be taken into account. Treatment of squamous cell carcinoma of the head and neck depends on the stage of the tumour. In the initial stages, a single treatment of surgery or radiotherapy may be sufficient. In contrast, unimodal treatments often fail in advanced stages. Despite correct treatment with conventional surgery and radiotherapy, the rate of loco-regional recurrence is approximately 50% to 60% and the rate of metastasis is between 20% and 40% (6). Among the major causes of morbidity and mortality are local invasion and regional lymphatic spread, metastases are considered a less frequent cause.

The objective of this research was to analyze type, characteristics, treatment and evolution of oral benign or malignant neoplasm or presence of precancerous lesion in a sample of the Andalusian population (Spain) derived from the Oncology Rehabilitation Hospital Unit during a period of 20 years.

## Material and Methods

An observational retrospective descriptive study was carried out during the years 1991 and 2011 analyzing the type, characteristics, treatment and follow-up of oral benign or malignant neoplasm or presence of precancerous lesion. The sample consisted of patients whose tumour had previously been removed and who had been referred to the Prosthetic Rehabilitation Department of the Oral and Maxillofacial Surgery Unit of the Universitary Hospital “Virgen del Rocío” (Seville, Spain).

The inclusion criteria were patients whose underlying pathology was any type of benign or malignant neoplasm or presence of precancerous lesion, which after their treatment and during the period of time that comprised the present study would have been referred to the Oncology Rehabilitation Unit. Exclusion criteria were patients who had been rehabilitated prior to their pathology through implant prosthesis, that the rehabilitator treatment was contraindicated and those we were not able to obtain the information related to the studied variables.

The information obtained from patients included in the study was from the hospital clinical records of the Oncology Rehabilitation Unit and was entered into a data collection sheet. The variables studied were: sex, age, smoking habit, alcoholic habit, base pathology, tumour location, treatment performed and presence of recurrence.

Smoking was assessed in 4 categories, smoking 0-10 cigarettes per day, smoking 10-20 cigarettes per day, smoking 20-30 cigarettes per day and smoking more than 30 cigarettes per day.

For the evaluation of alcoholic habit was used the test of identification of alcoholic disorders (AUDIT) (7). Risk categories in the typical consumption table were: low-risk drinkers, ≤17 SDU (170g) for females and ≤ 28 SDU (280g) for males per week; Moderate-risk drinkers, > 17 SDU for females and > 28 SDU for males per week; And high-risk drinkers, > 28 SDU for females and > 42 SDU for males per week (8).

The present study was authorized by the Ethics Committee of the University of Seville, and the patients signed their consent for their clinical data to be used for scientific purposes, although the patients could not be identified. Statistical analysis of the variables studied was performed using the SPSS program. A chi-square test (for the study of the distribution of the different variables in the sample) and univariate and multivariate logistic regressions (to identify risk factors for oncological pathology) were carried out.

## Results

Of the initial analyzed sample of 60 patients, only 45 patients met the inclusion criteria. Of the final sample analyzed, 31 subjects were men (68.9%) and 14 women (31.1%) (*p* = 0.0169). The mean age of the sample was 57 ± 13.83, being more frequent the presence of head and neck cancer in people over 50 years (73.3%) compared to people under 50 years of age (26.7%) (*p* = 0.0169). The different analyzed variables disaggregated by age are found in  Table 1.

**Table 1:**
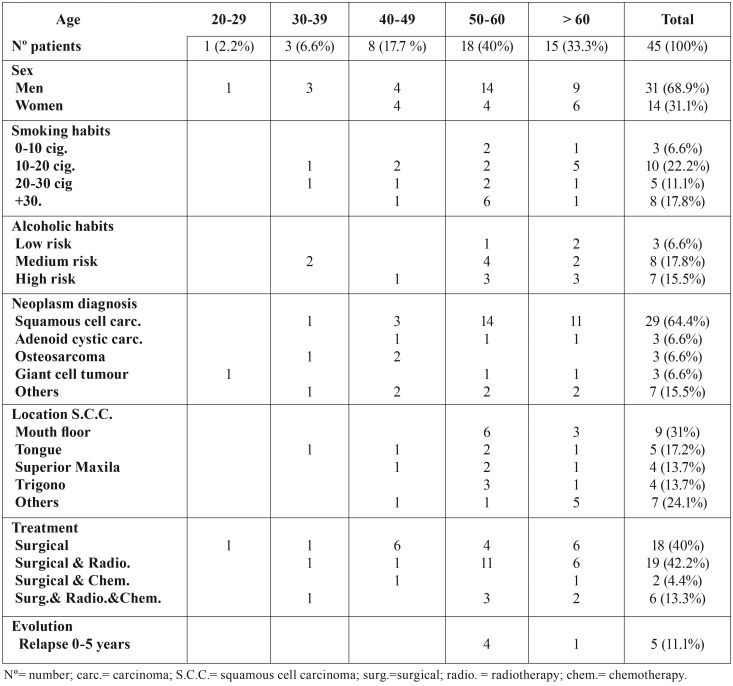
Analysis of studied variables by age.

Of the total sample of 45 patients, 57.8% had a history of smoking, of which 6.6% were smokers of 0-10 cigarettes per day, 22.2% were smokers of 10-20 cigarettes per day, 11.1% were smokers of 20-30 cigarettes a day and 17.8% smoked more than 30 cigarettes a day.

In relation to alcoholic habits, of the total sample studied, 40% had a history of alcoholic habits, 6% were prudent drinkers, 17.8% were moderate drinkers and 15.5% were excessive drinkers.

The most common pathology was epidermoid carcinoma (64.4%). Among the pathologies found less frequently were ameloblastoma, odontogenic keratocystic tumor, rabdiomyosarcoma, leiomyosarcoma and cavum lymphoepithelioma, with a respective frequency of 2.2%.

The most frequent localization found on squamous cell carcinoma was floor of the mouth (31%), tongue (17.2%), maxillary maxillary (13.7%) and retromolar trigone (13.7%) ( Table 1). Among the less frequent locations we could found were hard palate (6.8%), mandible, gingiva, amygdala, nasal fossa and soft-uvula palate (3.4%) respectively.

The most frequent treatment modality of the sample studied was surgery with postoperative radiotherapy (42.2%), followed by surgery as the only treatment (40%) and only 13.3% of the cases were performed after surgery a combination of radiotherapy with chemotherapy.

Of the sample studied, all patients had a minimum follow-up of 5 years, during this period only 5 patients in the sample studied presented recurrence of tumor pathology (11.1%), being more frequent in patients with a range between 50-60 years (80%). Of the 33 patients that could be evaluated during a period of 10-15 years, only 3 patients presented tumour recurrence (9%). With a period of 10-15 years, 20 patients could be evaluated and none of them had relapsed. A total of 9 patients with a follow-up greater than 15 years could be evaluated, of which only 1 patient had recurrence of oral tumour (12.5%).

The univariate logistic regressions showed a statistically significant association for the independent variables, age (*p* = 0.0169), sex (*p* = 0.0476), smoking (*p* = 0.0019) and alcoholic habit (*p* = 0.0106) with the dependent variable squamous cell carcinoma ( Table 2, Table 3)

**Table 2:**
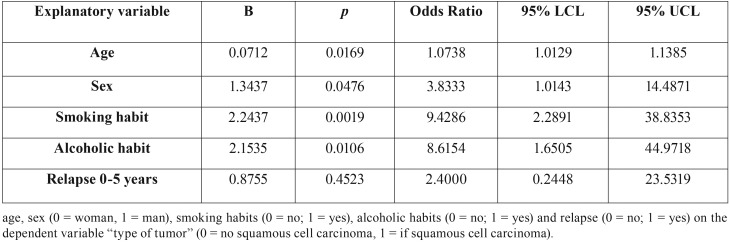
Univariate logistic regressions of the association of independent variables.

**Table 3:**
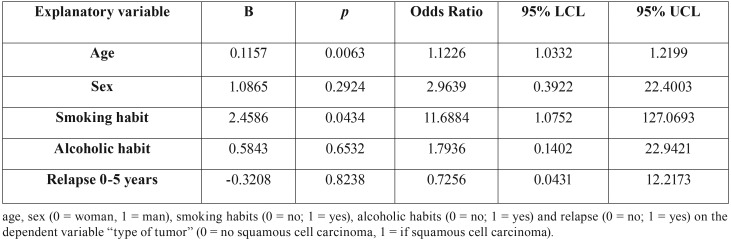
Multivariate logistic regressions of the association of independent variables.

The multivariate logistic regression according to the independent variables, age, sex, smoking habit, alcoholic habit and relapse 0-5 years, with the dependent variable presence of squamous cell carcinoma, only continued to show a statistically significant association for the variables age (*p* = 0.0063) and smoking habit (*p* = 0.0434).

## Discussion

The most common histology related to oral neoplasm was squamous cell carcinoma or epidermoid. Considered the malignant tumour of epithelial origin more common of the oral cavity. It is more frequent in men and the age of onset is usually between 50 and 70 years. Presence in children under 30 years of age is rare, although an increase in women younger than 30 years has been observed without being related to smoking habit (1,2,9-11).

In industrialized countries, the ratio between male and female is 2: 1, because the most important risk factors, tobacco and alcohol, have been more established in men. This proportion is changing because women, in recent years, are acquiring largely these habits. This fact reveals that 51% of patients from the Unit of Prosthetic Rehabilitation of the University Hospital Virgen del Rocío were female.

Krishna *et al.* (12) studied the prevalence of oral neoplasm in Asian countries between the years 2000 and 2012 and found an average age between 51-55 years. This is in agreement with our results where 40% of the patients attended at the unit had ages between 50-60 years.

Among the etiological factors of oral cavity neoplasm are smoking and alcohol consumption (13). Kuriakose *et al.* (14) observed in their study a small percentage of patients less than 45 years of age who did not smoke or drink and had epithelial dysplasias at the oral level. In our study, 57.8% of the patients studied had a history of smoking, although only 17.8% smoked more than 30 cigarettes per day. In relation to the alcoholic habit, 40% of the total sample had history, where only 15.5% were excessive drinkers.

The role of alcohol as an independent predisposing factor in oral neoplasm has been discussed in the literature (15). On the contrary, it is obvious that alcohol increases the risk of cancer in a patient with regular tobacco consumption; both factors have a carcinogenic and synergistic effect (16). The results of our study are congruent with those found in the literature, univariate logistic regressions found a statistically significant association between smoking (*p* = 0.0019, OR = 9.4), alcoholic habit (*p* = 0.0106, OR = 8.6) and suffer from epidermoid carcinoma. However, in the multivariate logistic regression only the smoking habit continued to present a statistically significant association (*p* = 0.0434; O.R = 11.6884).

Sanchis *et al.* (17) collected information on the prevalence of oral cancer and found that 38.8% appeared in the tongue (22.5% in the initial two thirds of the tongue), 26% in the floor of the mouth, 9.5% In the oral mucosa, 8.9% in gingiva, 8.8% in palate and 8% in the retromolar trigone and the tonsillar pillar. Similarly, Bodner *et al.* (18) in a review of 186 cases collected over 20 years of squamous cell carcinoma found that 38% were on the tongue, followed by the floor of the mouth with 26%. This does not match with our results, since a higher prevalence of squamous cell carcinoma was found in the floor of the mouth (31%).

The treatment of cancer in the oral cavity is mainly based in resective surgery, which usually involves an important anatomical alteration in the development of certain basic functions for the daily life of the patient, such as speech and chewing. In case the patient has received radiotherapy, other alterations may appear, such as damage of the patient’s own mucosa, xerostomia or possible dental caries. All this induce in the oral cavity an altered anatomical configuration that affect significantly the quality of life of the patients (19,20).

Fletcher *et al.* (21) in 1970 already published the first evidence of the benefit of combine radiotherapy and surgical treatment in the treatment of oral cavity carcinoma. This led to an in-depth study of this modality of treatment showing an increase treatment success with the application of postoperative radiation therapy. This combination therapy is mainly applied in patients with a high risk of relapse or metastasis that ha to be treated with more aggressive treatment (22).

Berneir *et al.* (23) performed a randomized study of 334 patients with stage III or IV stage III or IV cancer. They observed a 5-year survival greater in the combined treatment group of radiotherapy and postoperative chemotherapy than in the group of post-surgical radiotherapy. Although a greater adverse effects were found such as severe xerostomia, muscular fibrosis and mucosal necrosis. In our study, 13.3% of the patients in the sample received a combined treatment of radiotherapy and chemotherapy.

Licitra *et al.* (24) suggest that neoadjuvant chemotherapy may play an important role in the preservation of function and thus avoid radiotherapy for younger patients with oral cancer. However, the American Society of Clinical Oncology (ASCO) at the annual meeting in 2012 presented data in which it sees no advantage over neoadjuvant chemotherapy prior to the combination of chemotherapy and radiotherapy (25).

The survival of cancer today is a reality, although the values may vary depending on the type of injury and the time of diagnosis. In our unit, 11% of patients suffered relapse in the first 5 years. Of the 9 patients with a follow-up period of more than 15 years, 8 patients remained free of disease during this period (88.9%). The difference in the total number of patients per follow-up period is due to the fact that since the treatment date, this was the time that it was possible to count. Gastrom (26) in his 2005 study assessed the follow-up of 107 patients for 25 years, and observed an average survival of 16 years after the treatment of the underlying pathology.

## Conclusions

Epidermoid carcinoma is the most frequent tumour in the oral cavity, mainly found in the floor of the mouth, where advanced age and smoking are confirmed as associated risk factors.
